# Effects of a Dietary Multi-Mineral Bolus on Udder Health in Dairy Cows: A Clinical Assessment

**DOI:** 10.3390/vetsci11120621

**Published:** 2024-12-04

**Authors:** Jacopo Guccione, Maria Chiara Alterisio, Sergio Esposito, Giovanni D’Onghia, Sebastiano Tinelli, Antonio Di Loria, Beatrice Mercaldo, Alessandro Vastolo, Paolo Ciaramella

**Affiliations:** 1 Department of Veterinary Medicine and Animal Productions, University of Study of Napoli Federico II, Via Federico Delpino 1, 80137 Napoli, Italy; 2 Independent Researcher, Mottola Town, Taranto, 74017 Puglia, Italy; 3 Public Veterinary Health and Veterinary Assistance Service—Area A, Mottola Town, Taranto, 74017 Puglia, Italy

**Keywords:** mastitis, minerals, trace elements, copper, iodine, cobalt, selenium

## Abstract

The clinical effects on the udder health of several trace elements—copper, iodine, cobalt, and selenium—contained in an intraruminal slow-release bolus were explored for the first time. Fifty-four dairy cows received the bolus (treated group, TG), while fifty-three were left untreated (control group, CG). Monthly composite milk samples were collected from 30 to 300 days in milk to measure somatic cell count (SCC); milk production was also recorded on the same days. Cows with SCC > 200 × 10^3^ cells/mL were considered as affected by mastitis (with or without clinical signs). The effects on udder health were evaluated using several clinical indices employed for mastitis monitoring. The TG cows had a higher average daily milk yield than CG (*p* < 0.001), as well as a lower overall daily average of SCC (*p* < 0.0001). Fewer overall mastitis cases were detected in TG than in CG (*p* < 0.0001), although no significant differences were observed in new or cured mastitis cases. Finally, fewer failures of existing mastitis to cure cases were detected in TG as compared to CG (*p* < 0.0001), as well as fewer chronic mastitis cases (*p* < 0.0001). By looking at the clinical findings, some potential benefits on udder health might be hypothesized; nevertheless, additional studies are necessary to confirm these encouraging results.

## Simple Summary

Nutritional status has a pivotal role in animal health and production. Unfortunately, the animal’s trace mineral status is often overlooked until an animal’s performance falls below expectation or illness is detected; therefore, data exploring the effects of supplementation on udder health are still incomplete. Hence, this study aimed to assess the impact of trace elements—copper, iodine, cobalt, and selenium—delivered via a slow-release bolus on dairy cow udder health. The administered product might have contributed to promoting a reduction in the overall number of mastitis events, limiting the duration of the disease process, leading to a decrease in overall severity, and an increase in total milk production. Considering the observed effects, its use in an integrated approach that includes correct clinical strategies for mastitis prevention management might be supposed. Further studies involving a larger sample size, as well as considering the metabolic and trace element status of enrolled animals, should be performed to confirm the hypotheses.

## 1. Introduction

Mastitis is a widespread and expensive issue for the entire dairy food chain with significant effects on udder health, milk yield (MY), milk quality, and overall farm profitability [1]. The most common cause of mastitis is represented by bacterial infections, which can result in decreased milk production, drug treatment, veterinary costs, quarters lost, and even early culling of the animals in severe cases [2]. Traditional clinical care of mastitis still mainly relies on the administration of antibiotics; however, this method has led to the developing problem of antimicrobial resistance over time, posing a huge global threat to both animal and public health [3,4]. As a result, developing alternate methods to avoid and control this issue becomes crucial [3,4]. The scientific community [5] and several international agencies [6], authorities [7] and organizations [8] have recently emphasized the need to reduce antibiotic use in treating infectious diseases in livestock, especially in cattle, to combat the rising issue of antibiotic resistance in farms. This necessitates a comprehensive strategy that incorporates illness prevention, appropriate clinical management of animals, and the use of complementary and alternative techniques of treatment based on natural therapies, homeopathic medicines, and nutritional supplementations [8,9].

The role of individual trace minerals, such as copper (Cu), iodine (I), selenium (Se), and cobalt (Co), in improving bovine immune function and health was already explored in previous studies, although their role is debated, making this field of research still genuine and exciting [10,11]. These micro-elements are essential for several physiological and biochemical processes that contribute to animals’ resistance against infections [10,11]. For example, enzymes like superoxide dismutase (SOD) that are involved in immunological responses and the defense against oxidative stress require a correct amount of copper to optimize their tasks [10], while iodine contributes to controlling some immunological and metabolic processes, being a pivotal component of the thyroid hormone [12]. Selenium regulates the activity of the glutathione peroxidase enzyme that preserves cells from oxidative damage observed during the inflammatory processes [13]. Finally, cobalt can be considered a vital component of vitamin B12, which is required for the synthesis of red blood cells and the maintenance of metabolic health [14]. Several studies in the literature demonstrated that adequate supplementation of trace minerals can enhance immune responses, reduce oxidative stress, and increase the resilience of dairy cows to infections, including mastitis [10,15,16,17]. For example, selenium supplementation was correlated with lower somatic cell counts (SCC) in milk and improved udder health due to a reduction in the severity of the problem [15]; copper and iodine were instead associated with a reduced incidence of mastitis because of an observed enhancement of the immune function [16,17]. Despite the promising findings reported for trace mineral supplementation, their optimal amounts and combinations for preventing and managing mastitis, even avoiding excesses and deficiencies leading to adverse health effects, remain areas of active research [10], because the exact mechanisms by which they influence immune function and their synergistic effects are still not clear [18,19].

Based on the previous statement, this study aims to explore for the first time the effects on udder health and milk production of dairy cows, of a multi-mineral dietary supplement contained in a slow release intraruminal bolus, which includes copper, iodine, cobalt, and selenium.

## 2. Materials and Methods

### 2.1. General

This current study was performed in spring 2022 (from March to June) and involved 110 pluriparous (≥2 lactations), Holstein Friesian, dairy cows housed in a free-stall barn placed in the Puglia Region (Southern Italy).

All the procedures performed for data collection were part of a routine program unrelated to the study. The commercial bolus was bought by the farmer and independently administered to his animals for different purposes. Milk samplings were performed during the routine mastitis monitoring program regularly practiced by the local veterinary practitioner regardless of the study. All the clinical procedures performed during the routine mastitis monitoring program abided by the common good clinical practices [20]; moreover, the farmer was previously informed and agreed about the purposes and methods of the present investigation; written approval was also received. No additional interventions, modifications to standard practices, or new procedures were introduced for this investigation. Our research involved only the analysis of clinical data provided by the veterinarian and the farmer, and clinical data, not the animals themselves, were the focus of the analysis. Given the previous statements, approval by the Ethical Animal Care and Use Committee of the University of Napoli Federico II was not requested.

### 2.2. Farm and Animals

The selected farm was casually extracted within a group of 6 regularly requesting consultancy services at the Veterinary Teaching Hospital—Didactic Mobile Clinic Service of the Department of Veterinary Medicine and Animal Production of Napoli (Italy). The inclusion criteria to select the farm were as follows: (i) a similar herd size (~500 milking dairy cows, consistent along the year); (ii) a feeding system based on total mixed ration, given two times/day; (iii) the presence of a regular monitoring program for mastitis (including at least monthly milk sampling for SCC and data analysis); and (iv) housing and an overall management system respecting the minimum welfare standard for dairy cows. No strict criteria were applied for bulk milk somatic cell count (BMSCC) or mastitis incidence.

The selected farm was characterized by a herringbone parlor, and animals were milked twice a day. A 12-month average (µ**_x_**) of 335.3 ± 15.6 days in milk [DIM, ± standard deviation (SD)], overall µ**_x_** MY/head of 9448.7 ± 23.2 (kg ± SD), and BMSCC values of 413.2 × 10^3^ ± 203.2 × 10^3^ (cell/mL ± SD) have been recorded at sampling time. Milking cows were kept in two barns of ~600 m^2^ (~15 m × 40 m) characterized by solid, grooved concrete floors both in the walking and feeding alleys (cleaned five times a day with an automatic scraper). Cows abruptly dried off when they produced <10 L/day and were instead kept in a common paddock with a lying area consisting of a roofed deep straw yard area (~600 m^2^, cleaned once a week). Free access to the protected water trough was always guaranteed for all the animals’ categories (dried-off and milking cows), and they received three different rations (for dry-off, early lactation, and mid-late lactation phases). Details are provided in the Supplementary File S1 (Table S1).

### 2.3. Study Design and Mastitis Management

For this prospective and observational clinical trial, 54 cows were enrolled in the treated group (TG) and received two intraruminal slow-release boluses of a dietary multi-mineral feed at dry-off, as per label instructions (DalmaGlass SMR^®^, FATRO IBÉRICA S.L, Barcelona, España). Each bolus contained the following trace elements: copper oxide—13.4 g; anhydrous calcium iodate—1.0 g; cobalt carbonate—0.5 g; sodium selenite—150 mg. The release time of the trace elements was six months, as declared by the manufacturer. Another 53 cows were left untreated and served as the control group (CG).

The study population was selected based on the following criteria: (i) classified as healthy according to historical data, specifically a composite SCC value (pool of the four quarters) of less than 200 × 10^3^ cells/mL in the last three samples before dry-off, and no diseases or disorders in the last three months of the previous lactation; (ii) having no other diseases except mastitis, whether clinical (CM) or subclinical (SCM) at enrollment (hereby defined as mastitis). For this reason, each animal underwent a thorough clinical examination as described by Jackson and Cockcroft [21], with particular attention to the teat and udder at dry-off [22,23].

To retrospectively form two homogeneous groups and prevent selection bias arising from significantly different milk production levels, the overall µ**_x_** MY/head from the previous lactation was compared between the TG and the CG. Animals that met the selection criteria were excluded and replaced until there was no statistically significant difference in MY from the previous lactation between the two groups.

Throughout the study, any animal showing signs of mastitis (CM or SCM) was managed and/or treated by the practitioner using a routine protocol agreed upon with the farmer, regardless of the study. This protocol was not modified by the investigators and included the following:

- Upon notification by the milkers who regularly performed a macroscopic evaluation of the udder and milk, or following the analysis of monthly SCC results, all quarters of the reported cows were tested using the California Mastitis Test (CMT). The scoring system used was negative, trace, +1, +2, and +3 [24].
- In cases of SCM (CMT ≥ +1), the milk from quarters with high SCC was discarded for at least three consecutive days (6 milkings) after intramuscular administration of oxytocin to ensure complete emptying (Neurofisin^®^, Fatro Industria Farmaceutica Veterinaria S.p.A., Ozzano dell’Emilia, Italy). The other quarters were milked as usual. If the CMT was still positive at the end of the third day, milk was collected and sent to a certified laboratory for bacteriological culture (BC) and antibiogram testing. All quarters of milk samples were aseptically collected as described by the National Mastitis Council [25] for dairy cows. Complete emptying and discarding were continued until results were available (~48 h later). The correct antibiotic was chosen according to EMA [6] guidelines, and the withdrawal periods specified on the label were respected for the milk from all quarters of the udder.
- In cases of CM (presence of clinical signs at quarter or milk level), milk from the affected quarter was immediately collected for BC and antibiogram testing, while the other quarters were milked as usual if negative at the CMT. While awaiting results, complete emptying and discarding were performed as described for SCM. The process for the antibiotic selection and the milk withdrawal decision was also the same as for SCM.

Additional details regarding BC results are reported in Supplementary File S2 (Table S2).

### 2.4. Assessment of the Clinical Efficacy

The effects on udder health were assessed using the routine herd improvement data monthly recorded from one to ten months after calving and including MY and composite milk samples for SCC assessment (pool of four quarters). The latter were monthly collected by the diagnostic service of the Italian Breeders’ Association, while MY was recorded by the investigators on the same day of SCC sampling using dedicated farm software (Si@allEvA^®^, Italian Breeders’ Association, Rome, Italy, https://www.sialleva.it/ (accessed on 1 November 2024)). As suggested by Bradley et al. (2012), a threshold of SCC > 200,000 cells/mL was used to define cows affected by mastitis (CM or SCM) and those not affected (potentially affected by intramammary infection or healthy). The proposed classification was essential to employ the clinical mastitis monitoring indices suggested by Bradley et al. [26], which included the following: (i) overall and (ii) monthly daily µ**_x_** MY (data expressed in liters, L), (iii) µ**_x_** peak of production (data expressed in liters), (iv) monthly persistency of production (proportion of milk lost in the current sampling compared to the previous one), (v) overall and (vi) monthly mastitis cases (proportion of animals with SCC > 200,000 mL), (vii) overall and (viii) monthly new mastitis cases (proportion of cows with SCC > 200,000 cell/mL in the current sampling having SCC < 200,000 cell/mL in the previous one), (ix) overall and (x) monthly cured mastitis (proportion of cows with SCC > 200,000 cell/mL in the current sampling having SCC < 200,000 cell/mL in the following one), (xi) overall and (xii) monthly failure of existing mastitis to cure (proportion of cows with SCC > 200,000 cell/mL in the current month still having SCC > 200,000 cell/mL in the following one), and (xiii) overall and (xiv) chronic mastitis cases (proportion of cows with SCC > 200,000 cell/mL in at least two of the last three samplings).

### 2.5. Statistics

For the current investigation, data on MY and SCC were analyzed at the individual animal level. The sample size was determined based on Friedman’s [27] indications, assuming a correlation analysis with a power level of 0.80, a two-tailed significance level of 0.01, and an effect size of 0.45 (minimum required sample size = 50 cows per group). Additionally, through dedicated software, a retrospective power analysis was also performed (G*Power^®^ 3.1.9.7, 2024, Heinrich-Heine-Universität, Düsseldorf, Germany). The minimum number of measurements was obtained by the overall number of chronic mastitis cases because the smallest of all considered (i.e., with TG = 365 and CG = 344 measurements). An effect size of 0.30, associated with a two-tailed significance level of 0.05 and an achieved power of 0.98 (1-β error probability), was detected.

All data were analyzed using standard descriptive statistics and reported as absolute numbers, percentages, µ**_x_** ± SD, medians (Me), and interquartile range (Q1–Q3) (IQR1-3). Shapiro–Wilk tests, normal probability plots, and histograms were used to assess data distribution and normality. For continuous variables (MY and SCC), mean differences were explored using the Mann–Whitney U test. As described previously, several clinical indices were calculated to evaluate the clinical efficacy of the dietary mineral supplement, following the guidelines of Bradley et al. [26] that suggest the use of variables as categorical data for calculation (i.e., presence or absence of mastitis). A χ^2^ test and contingency tables were employed to define significant differences between expected and observed frequencies of categorical data, while Fisher’s exact test was applied in cases of low expected frequencies (<5). *p*-values < 0.05 were considered statistically significant.

As described by Monaghan et al. [28], the Relative Risk (RR) was calculated to quantify the likelihood of an effect (positive or negative) occurring in the TG compared to the CG; this was applied only for categorical variables showing significant differences between the two groups. The formula used was {RR = [a/(a + b)]/[c/(c + d)]}, where a = number of exposed animals with the event; b = number of exposed animals without the event; c = number of unexposed animals with the event; and d = number of unexposed animals without the event. All statistical analyses were performed using SPSS software (Version 29.0.1.0, Chicago, IL, USA).

## 3. Results

### 3.1. Farm and Animals

Regarding the enrollment time, all cows resuming milking during the springtime were included to ensure good homogeneity of the sampling population (µ**_x_** lactation number, TG = 2.7 ±1.4 and CG = 3.0 ±1.2). Indeed, an extension of recruitment time would have produced a population even more, overtime, distributed and without the possibility of reducing possible seasonal influences on the results observed. The overall µ**_x_** MY/head in the previous lactation was 6096.0 ± 47.7 (kg ± SD) and 6125.5 ± 32.3 for TG and CG, respectively (*p* = 0.07).

The number of cows monthly sampled suffered from a progressive reduction during lactation in both groups. The reasons for this progressive decrease in the number of animals were mainly due to (i) planned replacement, (ii) spontaneous shorter lactation, and (iii) culling because of other diseases’ appearance. Details regarding the number of cows monthly sampled were reported in Table 1.

### 3.2. Samplings and Milk Yield

During the investigation, n°1846 routine herd improvement recordings data were collected that included both SCCs (n°923) and MYs (n°923). Out of them, n°473 data belonged to the TG and n°450 to the CG. A significant difference was observed between the overall daily µ**_x_** MY between the two groups (TG = 38.7 L ± 9.5 vs. CG = 37.1 L ± 8.8, *p* < 0.001), while no differences were observed for the monthly daily µ**_x_** MY between the two groups as well as for the µ**_x_** pick of production and monthly persistency of production. Further details on production data are provided in Table 1.

### 3.3. Udder Health Status

A significant difference was observed regarding the overall daily µ**_x_** of SCC (cells/mL × 10^3^) between TG [Me = 67.0 × 10^3^, IQR1-3 = 6.0 × 10^3^–10,819.0 × 10^3^] and CG (Me = 86.0 × 10^3^, IQR1-3 = 9.0 × 10^3^–25,966.0 × 10^3^) (*p* < 0.0001). Moreover, an additional difference was observed between the two groups for the monthly µ**_x_** of SCC at 150 (µ**_x_** = 407 × 10^3^, Me = 54.0 × 10^3^, IQR1-3 = 11.0 × 10^3^–10,819.0 ×10^3^ vs. CG, Me = 83.0 × 10^3^, IQR1-3 = 18.0 × 10^3^–16,016.0 × 10^3^, *p* < 0.05) and 270 DIM (TG, Me = 23.0 × 10^3^, IQR1-3 = 43.0 × 10^3^–3815.0 × 10^3^ vs. CG, Me = 93.0 × 10^3^, IQR1-3 = 24.0 × 10^3^–2308.0 × 10^3^, *p* < 0.05). Further details on SCC are provided in Table 2.

**Figure 1:**
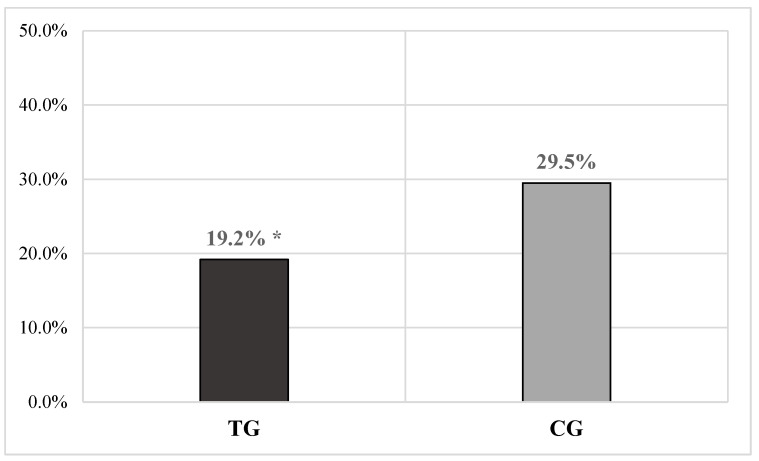
The bar graph illustrates the total number of mastitis cases in a dataset of cows receiving a complementary feed via an intraruminal slow-release bolus (TG = 473 measurements) compared to a control group not receiving the bolus (CG = 450 measurements). A significantly lower number of mastitis cases was observed in the TG compared to the CG (* *p* < 0.0001). Caption: TG = treated group; CG = control group; % = percentage.

A significantly lower overall number of mastitis cases was observed in the TG compared to the CG (TG = 91/473 or 19.2% vs. CG = 133/450 or 29.5%; χ^2^ = 13.226, *p* < 0.0001) (Figure 1), with a relative risk (RR) of 0.66 in the TG and 1.53 in the CG for this variable.

Moreover, a significant difference was observed between the two groups for the monthly cases of mastitis at 60 (TG = 4/54 or 7.4% vs. CG = 14/53 or 26.4%, χ^2^ = 6.9064, *p* < 0.01), at 150 (TG = 9/54 or 16.6% vs. CG = 17/50 or 34.0%, χ^2^ = 4.16, *p*< 0.05), and at 270 DIM (TG = 5/32 or 15.6% vs. CG = 12/32 or 37.5%, χ^2^ = 3.9249, *p* < 0.05).

No significant intergroup differences were instead observed for the indexes overall (TG = 39/419 or 9.3% vs. CG = 39/397 or 9.8%) and monthly new mastitis cases, as well as for the indexes overall (TG = 46/419 or 11.0% vs. CG = 48/397 or 12.1%) and monthly cured mastitis cases.

**Figure 2:**
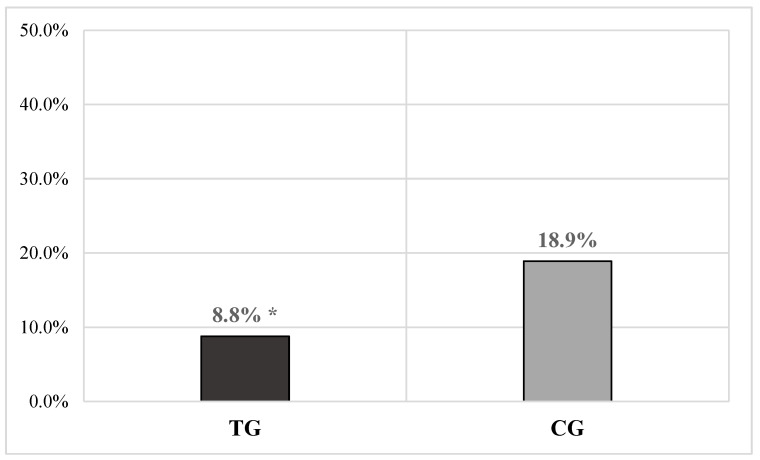
The bar graph illustrates the overall failure of existing mastitis to cure cases of mastitis cases in a dataset of cows receiving a complementary feed via an intraruminal slow-release bolus (TG = 473 measurements) compared to a control group not receiving the bolus (CG = 450 measurements). A significantly lower number of mastitis cases was observed in the TG compared to the CG (* *p* <0.0001). Caption: TG = treated group; CG = control group; % = percentage.

A significantly lower overall number of monthly failures of existing mastitis to cure cases was detected in TG compared with the CG (TG = 37/419 or 8.8% vs. CG = 75/397 or 18.9%, χ^2^ = 17.426, *p* < 0.0001) (Figure 2), with a RR of 0.47 in the TG and 2.14 in the CG.

Moreover, a significant monthly difference was observed between the two groups for the same parameter at 60 (TG = 9/54 or 16.7% vs. CG = 0/53, χ^2^ = 10.165, *p* < 0.001) and 90 DIM (TG = 9/54 or 16.7% vs. CG = 2/53 or 3.8%, χ^2^ = 6.441, *p* < 0.05).

**Figure 3:**
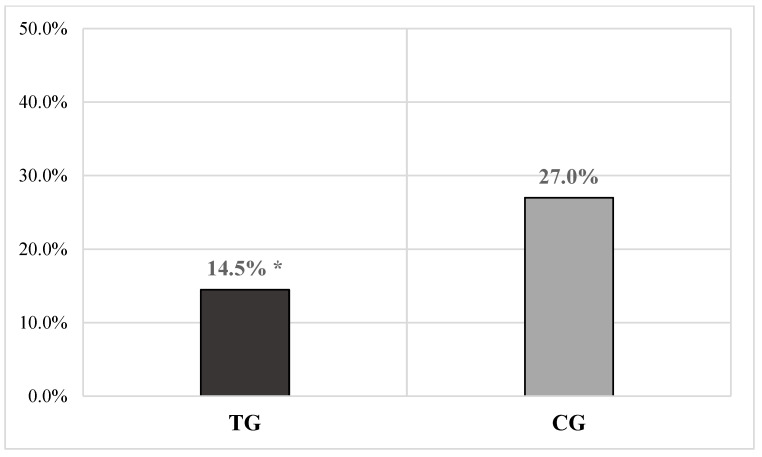
The bar graph illustrates the overall chronic mastitis cases in a dataset of cows receiving a complementary feed via an intraruminal slow-release bolus (TG = 473 measurements) compared to a control group not receiving the bolus (CG = 450 measurements). A significantly lower number of mastitis cases was observed in the TG compared to the CG (* *p* < 0.0001). Caption: TG = treated group; CG = control group; % = percentage.

A significantly lower overall number of chronic mastitis cases was detected in TG as compared with the CG (TG = 53/365 or 14.5% vs. CG = 93/344 or 27.0%, χ^2^ = 16.961, *p* < 0.0001) (Figure 3), with a RR of 0.54 in the TG and 1.82 in the CG. Moreover, a significant monthly difference was observed between the two groups for the same parameter at 90 (TG = 3/54 or 5.5% vs. CG = 14/53 or 26.4%, χ^2^ = 8.901, *p* < 0.01) and 120 DIM (TG = 3/54 or 5.5% vs. CG = 12/53 or 22.6%, χ^2^ = 6.640, *p* = 0.01). Further details of the clinical index results are provided in Table 2.

## 4. Discussion

To the best of the authors’ knowledge, this study is the first to describe the effects on the udder’s health of dairy cows of a multi-mineral dietary supplement contained in a slow-release intraruminal bolus, which includes copper, iodine, cobalt, and selenium. Estimating the effectiveness of supplements as a support for the prevention and management of mastitis on farms is a stimulating challenge for bovine medicine [29]. This is especially relevant as the search for alternative methods to improve the control of this disease without resorting to antibiotics is a global priority [30]. This study fully addresses the previous needs and adheres to the guidelines established by numerous international agencies, which unanimously indicate that the continuous and/or improper use of antibacterial molecules is an outdated practice and a genuine risk factor for the promotion of antibiotic resistance in livestock farms [8,31,32]. In this sense, the apparent paradox that arises from the need to have healthier animals on one hand and to minimize the therapeutic options on the other could represent an opportunity for the development of innovative, safe, and effective alternatives for the treatment and prevention of diseases. In order to contribute to the “One Health” objective, the scientific community has, therefore, the ethical obligation to collaborate to be part of novel strategies for disease control and prevention [8,31,32].

Nutrition plays a crucial role in the prevention and management of diseases in dairy ruminants, particularly mastitis; a well-balanced diet ensures the optimal functioning of the immune system, essential for counteracting infections and preserving udder health [33]. Fundamental nutrients, such as amino acids, vitamins (e.g., A, E, etc.), and trace minerals (e.g., copper, selenium, etc.), are indispensable for supporting the immune response and the overall health of the mammary gland [29]. The latter are essential trace elements for antioxidant defense, the functioning of the immune system (by modulating the effectiveness of systemic and local immune responses), and supporting production [10]. Although these elements seem to play a key role in protecting animal health and optimizing its production capacity, scientific knowledge about their mechanisms of action and clinical-therapeutic-prophylactic effects is often fragmented and contradictory [10,29]. These discrepancies can be explained by considering the variability of study conditions, administration methods, the nutritional status of the animals, and potential interactions with other nutrients in the diet, which can equally influence the outcomes of scientific trials [29,34].

Among the micro-elements contained in the slow-release bolus, the first to be mentioned is copper. From the literature, it can be learned that the recommended amount of this mineral in dairy cows varies from 5 to 20 mg/kg of DM [35], while toxic effects are observed when values exceed 40 mg/kg of DM in the diet [36]. Compared to other micro-elements, scientific literature highlights a primary role for copper in protecting udder health and animal welfare. Indeed, it is one of the most common metallic micronutrients able to ensure the structural and catalytic properties of some antioxidant enzymes (e.g., cytochrome-c oxidase, SOD), also having indirect antimicrobial properties associated with the oxidative destruction exerted on bacterial lipids, proteins, and DNA; this dual ability can be exploited to improve udder health management and prevent mastitis in dairy cows [37,38]. According to Reyes-Jara et al. [17], a copper concentration of 250 ppm was able to inhibit the in vitro growth of mastitis-causing microorganisms such as *Escherichia coli* (*E. coli*) and coagulase-negative *staphylococci*. Several studies have shown that dietary copper supplementation (20 ppm for 100 days in the treated group, compared to 6.5 ppm for 100 days in the control group) significantly reduced the intensity of clinical mastitis symptoms in dairy cows experimentally infected intramammarily with *E. coli* [39]. Gakhar et al. [37] observed a reduced incidence of post-partum mastitis in cows receiving copper supplementation compared to those that did not. Considering the above, the copper supplement employed in our study could be among the factors that contributed to a reduction in the overall incidence of mastitis in the GT (*p* < 0.0001) (Figure 1), as well as to strengthen the negative association between disease and exposure in the GT (the factor can protect against the disease) and the positive one in the GC (the lack of the factor can cause the disease) (GT-RR = 0.66 vs. GC-RR = 1.53) (Figure 1). Moreover, the overall reduction in clinical mastitis cases in the GT may have significantly influenced both the reduction in the overall average SCC values (*p* < 0.0001) and the increase in the overall average milk production in this group of animals (*p* < 0.001). However, it should be noted that the micro-element does not appear to have had any effect on either the overall likelihood of animals becoming infected and developing new cases of mastitis or on the overall cure rate.

Iodine, on the other hand, is the main constituent of thyroid hormones, including thyroxine (T4) and triiodothyronine (T3), and is predominantly present in the thyroid gland, which stores about 80% of the body’s total iodine [16]. The recommended amount of this mineral in dairy cows’ ration ranges from 0.5 to 5 mg/kg of DM [40], while toxic effects are observed when values exceed 20 mg/kg of DM in the diet [36]. Iodine is commonly absorbed in the rumen, from where it is transported in the body through specific plasma proteins [41]. Iannaccone et al. [16] reported an improvement in the short-term immune response efficacy (due to iodine’s ability to stimulate the activation of B lymphocytes, resulting in increased antibody synthesis, and the phagocytic activity of polymorphonuclear leukocytes) and a better oxidative state in cattle that received iodine administration; the study also demonstrated a lower SCC in infected cows that received an iodine supplement, compared to an untreated control group. However, the effects of iodine on milk production are unclear, and conflicting results have been observed in the literature. Some studies suggest that iodine is a limiting factor for milk production, while others report minimal variations after supplementation with different doses [16]. Looking at the results of our study, even this element might have been one of the factors that, together with the action of copper, might have contributed to the reduction in SCC; while considering the contrasting opinions in the literature regarding its effects on production, a significant action in this regard seems less likely.

Cobalt is a micro-element with a requirement in ruminants between 0.1 and 0.2 mg/kg of DM [14], and its dietary supplementation is associated with a maximum tolerable quantity estimated at 25 mg/kg of DM [36]. The functional role of cobalt is as a main component of cobalamin (vitamin B12), produced through fermentation by ruminal microbiota, provided that the concentration of cobalt in the ruminal liquid is above 0.5 mg/mL [42]. This vitamin participates as a coenzyme in two essential enzyme systems (methylmalonyl-CoA mutase and methionine synthase) for energy production from ruminal metabolism and is involved in the proper functioning of multiple metabolic reactions (e.g., metabolism of carbohydrates, fats, some amino acids, etc.) [14]; it also plays a role in the erythropoiesis process and the proper functioning of the nervous system [14]. High-yield dairy cows often have low efficiency in the production and absorption of vitamin B12, with levels varying significantly during lactation [43]. Cobalt deficiency is associated with increased susceptibility to disease development and reduced immune system function, as this mineral enhances the action capacity of neutrophils in sheep, and vitamin B12 deficiency, which results from it, can lead to a reduction in CD8+ Th lymphocytes and NK cells, thereby reducing the effectiveness of the immune system and increasing the likelihood of contracting diseases, especially of an infectious nature [44,45]. Considering these known biological functions, it could be hypothesized that supplementation of this micro-element might have further aided the proper functioning of the immune system, which in turn might have led to a significant overall reduction in the duration of mastitis in the GT compared to the GC, evidenced by (i) the reduction in overall cases of chronic mastitis (*p* < 0.0001) (Figure 3) and (ii) the decrease in the number of cows in whom the therapy was ineffective (*p* < 0.0001) (Figure 2). The observed results could suggest that the supplementation of this micro-element might also be among the factors that have contributed to improving the effectiveness of the management plan for mastitis applied by the holding. Even in this case, the findings seem to be supported by the RR values observed for the variable failure of existing mastitis to cure (GT = 0.47 vs. GC = 2.14) and chronic mastitis cases (GT = 0.54 vs. GC = 1.820). However, even in this case, it must be noted that the micro-element does not seem to have contributed to reducing the overall appearance of animals becoming infected and developing new cases of mastitis, nor to the overall cure rate. Finally, regarding the effects on production, the results in the literature are also conflicting even for cobalt. Kincaid and Socha [46] observed no effect of cobalt supplementation on the quantity and quality of milk produced by cows, while Kincaid et al. [47] described a linear increase in milk production in multiparous animals when cobalt was given in supplementary doses of 1 mg/kg, but no similar effects were observed in primiparous cows. This uncertainty of results did not allow the authors to confirm a direct action of the element on milk production.

Of the minerals contained in the slow-release bolus, selenium is probably the one that has received the most attention in bovine medicine. Selenium plays a crucial role in managing herd health and welfare; deficiencies can predispose cows to reduced fertility, retained placenta, and increased incidence of mastitis and metritis [15]. The dietary requirement for selenium in dairy cattle ranges from 0.1 to 0.3 mg/kg of DM [15], while toxicity can occur with 5 mg/kg of DM in the ration [36]. This element is known for its antioxidant, immunomodulatory, and antibacterial activities; it regulates key genes associated with the functioning of antioxidant enzymes [e.g., glutathione peroxidase (GSH-Px), catalase, SOD, and glutathione], and improves oxidative status and plasma levels of GSH-Px [15,48]. Several studies suggest that selenium supplementation is associated with an improvement in udder health. For example, Hoque et al. [49] demonstrated that cows receiving selenium supplementation intravenously were less prone to developing mastitis compared to those receiving only antibiotics. Ganda et al. [50] showed that the injection of trace elements, including selenium, reduced chronic mastitis cases in lactating cows, although it had no impact on the incidence of clinically manifest cases requiring treatment. Another study showed a significant reduction in the incidence of mastitis during lactation in response to eight weeks of selenium supplementation in cows during the peripartum period [51]. Finally, Chen et al. [52] reported that low selenium levels can promote oxidative stress in the mammary gland, significantly increasing the number of apoptotic epithelial cells. Moreover, it has been demonstrated that a reduction in the prevalence of subclinical mastitis and, consequently, SCC was linked to an increase in GSH-Px activity in the blood of cows receiving selenium supplementation [48]. Considering the cited bibliographic references, even selenium administered in our study might have significantly contributed to the reduction of overall mastitis incidence in the GT (*p* < 0.0001) and the relative risk value in this group, as well as might have supported the proper functioning of the immune system, participating in the significant overall reduction in the duration of mastitis in the GT compared to the GC, evidenced by (i) the reduction in overall chronic mastitis cases (*p* < 0.0001) (Figure 3) and (ii) the decrease in the number of cows in which the therapy was ineffective (*p* < 0.0001) (Figure 2). Like cobalt, the outcomes seem to be supported by the RR values observed for the variable failure of existing mastitis to cure (GT = 0.47 vs. GC = 2.14) and chronic mastitis cases (GT = 0.54 vs. GC = 1.820). As previously mentioned for copper, in this case, the overall reduction in clinical mastitis cases in the GT may have significantly influenced the reduction of overall average SCC values (*p* < 0.0001) and, consequently, the increase in overall average milk production in this group of animals (*p* < 0.001). In the literature, the correlation between selenium administration and increased production in dairy cows is the subject of several studies, but the results are not entirely consistent and sometimes anecdotal [15]. Cook and Green [34] observed that milk production by the 100th day of lactation was significantly higher in cows supplemented with selenium, iodine, and cobalt; this study represents one of the few scientific pieces of evidence of a direct effect of selenium on milk production (although the authors cannot rule out an interaction between the micro-elements). Indeed, Respati et al. [53] clearly stated in their recent meta-analysis that selenium intake, in any form and type, has no relationship with milk yield, preventing the authors from supporting the idea of a direct effect even in their study. Finally, looking at the rest of the results obtained, selenium also does not seem to have had effects on the overall possibility of animals becoming infected and developing new mastitis cases nor on the overall cure rate.

After exploring the potential link between bolus and udder health, the authors would like to express some practical considerations related to the use of similar products. On one side, farms might benefit from its use as it simplifies the process of supplementing trace elements, providing a balanced mix of essential micro-minerals in a single product. This ensures that animals receive a consistent and appropriate dose, reducing the time and effort required to manage individual supplementation programs. Additionally, using a well-formulated product can minimize the risk of deficiency-related diseases, thereby reducing the need for emergency interventions and treatments [33]. The use of the product might contribute to increasing the farm’s profitability since healthy cows are more productive and require fewer expenses related to health [29,34,54]. On the other side, potential negative aspects are primarily related to the excessive and harmful intake of micro-elements; excessive intake of these trace elements can lead to toxicity and severely compromise the health and welfare of dairy cows [36]. Other negative aspects for operators might involve improper handling of the product or accidental exposure to high concentrations of trace elements with recognized human toxicity (e.g., cobalt, iodine), which can pose health risks [55]. Finally, the unnecessary use of such products can represent a significant financial burden for farmers that can additionally reduce milk yields and quality [29].

Finally, some limitations of the study mainly concerning the small number of samples as well as the lack of information regarding the metabolic and trace elements status of the enrolled animals need to be mentioned. As regards the first aspect, although the overall number of determinations gives solid information, the number of monthly recordings should be increased through further studies including a larger number of animals to provide greater statistical strength to the results. Regarding the second one, the absence of metabolic and micro-elements information does not allow for a comprehensive framework of the cows’ health status, which could have further supported the clinical findings and facilitated the interpretation of some of the results obtained.

## 5. Conclusions

This study aimed to assess the health effects on dairy cow udders of a group of trace elements—copper, iodine, cobalt, and selenium—contained in a slow-release commercial bolus. The administered product might have contributed to promoting a reduction in the overall number of mastitis events, limiting the duration of the disease process, and leading to a decrease in overall somatic cell counts and an increase in total milk production. However, despite these encouraging results, it should be noted that the administration had no effect either on the overall number of animals becoming infected and developing new cases of mastitis or on increasing the overall cure rate. Considering the observed effects, its use in an integrated approach that includes correct clinical strategies for mastitis prevention management might be supposed. Further studies involving a larger sample size, as well as considering the metabolic and trace element status of enrolled subjects, should be performed to complement the findings.

## Figures and Tables

**Table 1:** Routine herd improvement data on milk yield were to evaluate the clinical efficacy of a dietary mineral supplement delivered via an intraruminal slow-release bolus on the udder health of treated dairy cows. A separate group of cows, which remained untreated, served as the control group.

Production Data											
**MoS**		1	2	3	4	5	6	7	8	9	10
**DIM**		30	60	90	120	150	180	210	240	270	300
**Cows** (No)	**TG**	54	54	54	54	54	51	51	45	32	24
	**CG**	53	53	52	52	50	49	48	39	32	22
**µ_x_ MY** (L ± SD)	**TG**	39.6 (±10.9)	45.0 (±7.9) $	44.5 (±8.0)	41.2 (±9.0)	39.9 (±8.3)	38.1 (±8.0)	35.2 (±7.5)	32.9 (±8.8)	31.8 (±7.4)	30.4 (±7.8)
	**CG**	39.0 (±8.5)	42.8 (±9.1)	43.0 (±8.2) $	40.5 (±8.1)	37.8 (±6.6)	36.1 (±6.4)	33.2 (±6.8)	31.5 (±6.3)	28.2 (±6.0)	27.8 (±4.1)
**Me MY** (L)	**TG**	39.1	45.4	45.3	40.4	40.7	37.8	35.8	34.8	31.3	30.4
	**CG**	39.1	44.4	44.1	40.8	37.1	35.5	33.7	31.8	29.2	27.8
**MY IQR 1** (L)	**TG**	30.2	39.8	39.1	35.4	32.7	31.0	29.2	25.4	26.9	24.2
	**CG**	32.5	37.1	39.3	36.1	34.2	31.7	27.5	27.3	23.7	24.6
**MY IQR 3** (L)	**TG**	47.4	50.5	50.0	47.6	46.2	43.8	40.8	39.8	35.8	36.5
	**CG**	45.2	49.5	47.6	46.3	42.6	40.8	38.2	34.7	34.0	30.5
**PoP** (%)	**TG**	/	/	−2.0	−4.0	−3.0	−5.0	−8.0	−7.0	−3.0	−4.0
	**CG**	/	/	/	−6.0	−7.0	−5.0	−8.0	−4.0	−11.0	−2.0

Caption: MoS = months of sampling; DIM = days in milk; TG = treated group; CG = control group; No = number; µ**_x_** = average; MY = milk yield; Me = median; $ = peak of production; IQR 1 = interquartile range, 25th percentile; IQR 3 = interquartile range, 75th percentile; PoP = persistency of production from the peak; L = liters; SD = standard deviation; % = percentage.

**Table 2:** Routine herd improvement data on somatic cell count and clinical indexes used to evaluate the clinical efficacy of a dietary mineral supplement delivered via an intraruminal slow-release bolus on the udder health of treated dairy cows. A separate group of cows, which remained untreated, served as the control group.

Somatic Cell Count and Clinical Indexes											
**MoS**		1	2	3	4	5	6	7	8	9	10
**DIM**		30	60	90	120	150	180	210	240	270	300
**µ_x_SCC** *Cells/mL × 10^3^ (±SD)*	**TG**	631 (1579)	177 (531)	325 (1413)	367 (1080)	407 ^c^ (1541)	284 (869)	364 (1013)	239 (576)	184 ^a^ (401)	192 (229)
	**CG**	398 (893)	571 (1998)	307 (649)	304 (733)	793 ^d^ (2623)	553 (2067)	492 (946)	983 (4172)	418 ^b^ (793)	275 (286)
**Me SCC** *Cells/mL × 10^3^ (±SD)*	**TG**	61.0	34.0	47.5	53.0	54.0	66.0	107.0	107.0	93.0	95.0
	**CG**	73.0	72.0	50.0	58.5	83.0	84.0	88.0	119.0	132.0	173.5
**SCC IQR 1** *Cells/mL × 10^3^*	**TG**	12.0	8.0	6.0	11.0	11.0	15.0	16.0	12.0	24.0	18.0
	**CG**	35.5	20.5	26.5	37.2	51.7	43.0	55.5	72.0	77.5	107.0
**SCC IQR 3** *Cells/mL × 10^3^*	**TG**	7125.0	3041.0	10,072.0	7064.0	10,819.0	5956.0	6044.0	3768.0	2308.0	995.0
	**CG**	324.5	244.5	172.3	204.3	281	267.5	344.3	214.0	298.8	375.7
**Cases of mastitis** *(No)*	**TG**	15	4 ^c^	6	11	9 ^a^	12	11	11	5 ^a^	7
	**CG**	18	14 ^d^	12	13	17 ^b^	15	12	11	12 ^b^	9
**New monthly mastitis cases** *(No)*	**TG**	/	2	6	9	3	6	3	4	2	4
	**CG**	/	6	3	4	5	6	2	4	8	7
**Monthly cured mastitis cases** *(No)*	**TG**	/	12	4	4	5	3	4	4	3	2
	**CG**	/	10	5	3	2	7	5	5	4	5
**Failure to cure mastitis cases** *(No)*	**TG**	/	2 ^e^	0 ^a^	2	6	6	8	7	3	3
	**CG**	/	8 ^f^	9 ^b^	9	11	9	10	7	8	4
**Chronic mastitis cases** *(No)*	**TG**	/	/	3 ^c^	3 ^g^	6	9	9	11	7	5
	**CG**	/	/	14 ^d^	12 ^h^	13	13	11	11	10	9

Caption: MoS = months of sampling; Mo = monthly; DIM = days in milk; TG = treated group; CG = control group; No = number; MY = milk yield; µ**_x_** = average; Me = median SCC = somatic cell count; mL = milliliters; SD = standard deviation; IQR 1 = interquartile range, 25th percentile; IQR 3 = interquartile range, 75th percentile; Min = minimum; Max = maximum; ^a,b^: *p* < 0.05; ^c,d^: *p* < 0.01; ^e,f^: *p* < 0.001; ^g,h^: *p* = 0.01.

## Data Availability

The original contributions presented in this study are included in the article/Supplementary Material. Further inquiries can be directed at the corresponding author.

## Supplementary Materials

The following supporting information can be downloaded at: https://www.mdpi.com/article/10.3390/vetsci11120621/s1, Table S1: The table provides detailed information on the feeds included in the total mixed ration for dry-period, early lactation, and mid-late lactation stages. It also reports the overall trace element values provided by each ration. These formulations were developed by a veterinary specialist in ruminant nutrition employed by the farm. The total amounts of copper, iodine, cobalt, and selenium have been quantified by a specialized laboratory, which annually receives samples of the various feeds from the nutritionist; Table S2: The table summarizes the clinical udder health status based on overall bacteriological culture results. It presents data from assessments conducted according to the mastitis management protocol used on the farm. The table differentiates also cases with clinical signs (clinical mastitis, CM) and those without clinical signs (subclinical mastitis, SCM). The data are drawn from the comprehensive dataset of cows, including those receiving the complementary feed via an intraruminal slow-release bolus and those that did not.
