# Supplementary material for: Effects of a Dietary Multi-Mineral Bolus on Udder Health in Dairy Cows: A Clinical Assessment
Source: Vet Sci. 2024 Dec 4;11(12):621. doi: 10.3390/vetsci11120621 (PMC11680380; doi:10.3390/vetsci11120621)
Supplement: Supplementary file 1 [file vetsci-11-00621-s001.zip › Guccione et al._2024_Supplementary_file_S1.pdf]

SUPPLEMENTARY FILE S1

ORIGINAL RESEARCH

TITLE

Effects of dietary multi-mineral bolus on udder health in dairy cows: a clinical assessment

Jacopo Guccione [1], Maria Chiara Alterisio [1,*], Sergio Esposito [1], Giovanni D'Onghia [2], Sebastiano Tinelli [3], Antonio Di Loria [1], Beatrice Mercaldo [1], Alessandro Vastolo [1] and Paolo Ciaramella [1]

[1] Department of Veterinary Medicine and Animal Productions, University of Study of Napoli Federico II, Via Federico Delpino 1, 80137 Napoli, Italy
[2] Independent Researcher, Mottola Town, Taranto, 74017 Puglia, Italy
[3] Public Veterinary Health and Veterinary Assistance Service—Area A, Mottola Town, Taranto, 74017 Puglia, Italy
* Correspondence: mariachiara.alterisio@unina.it; Tel.: +39-0812536001

Table S1: The table provides detailed information on the feeds included in the total mixed ration for dry-period, early lactation, and mid-late lactation stages. It also reports the overall trace element values provided by each ration. These formulations were developed by a veterinary specialist in ruminant nutrition employed by the farm. The total amounts of copper, iodine, cobalt, and selenium have been quantified by a specialized laboratory, which annually receives samples of the various feeds from the nutritionist.

FEEDS INCLUDED IN THE RATIONS			
	**Dry-period** *(DM kg)*	**Early lactation** *(DM kg)*	**Mid-late lactation** *(DM kg)*
Mais silage	1.7880	5.9600	5.4400
Polyphite hay	3.7500	3.1234	3.5696
Alfalfa silage	-	2.7018	2.7018
Wheat straw	3.6800	0.2760	0.9200
Maize flour		5.8491	3.7539
Soybean flour	0.8891	3.2400	2.5200
Whole cottonseed	-	1.8320	0.9556
Wheat bran	-	2.1965	2.1965
Hulled sunflower seeds	0.7462	0.7462	0.9327
Barley flour	0.4441	0.8882	0.8882
Urea	-	0.0392	0.0294
Saponified fats	-	0.1910	-
Calcium carbonate	0.0399	0.2500	0.2196
Sodium bicarbonate	-	0.2500	0.2066
Dicalcium phosphate	-	0.0400	0.0388
Magnesium oxide	-	0.0700	0.0500
Sodium chloride	0.0392	0.0600	0.0490
Magnesium chloride	0.0187	-	-
Magnesium sulphate	0.0792	-	-
Water	-	0.0300	0.0300
**OVERALL TRACE ELEMENTS' VALUES PROVIDED BY THE RATIONS**			
	**Dry- period** *(mg/kg of DM)*	**Early lactation** *(mg/kg of DM)*	**Mid-late lactation** **(***mg/kg of DM)*
Cupper	7.83	9.85	9.97
Iodium	0.41	0.46	0.49
Cobalt	0.23	0.24	0.23
Selenium	0.14	0.15	0.16

DM=dry matter; Mg=milligrams; Kg=kilogram
