# Supplementary material for: Effects of a Dietary Multi-Mineral Bolus on Udder Health in Dairy Cows: A Clinical Assessment
Source: Vet Sci. 2024 Dec 4;11(12):621. doi: 10.3390/vetsci11120621 (PMC11680380; doi:10.3390/vetsci11120621)
Supplement: Supplementary file 1 [file vetsci-11-00621-s001.zip › Guccione et al._2024_Supplementary_file_S2.pdf]

## SUPPLEMENTARY FILE S2

## ORIGINAL RESEARCH

### TITLE

# Effects of dietary multi-mineral bolus on udder health in dairy cows: a clinical assessment

**Jacopo Guccione [1], Maria Chiara Alterisio [1,*], Sergio Esposito [1], Giovanni D'Onghia [2], Sebastiano Tinelli [3], Antonio Di Loria [1], Beatrice Mercaldo [1], Alessandro Vastolo [1] and Paolo Ciaramella [1]**

[1] Department of Veterinary Medicine and Animal Productions, University of Study of Napoli Federico II, Via Federico Delpino 1, 80137 Napoli, Italy
[2] Independent Researcher, Mottola Town, Taranto, 74017 Puglia, Italy
[3] Public Veterinary Health and Veterinary Assistance Service—Area A, Mottola Town, Taranto, 74017 Puglia, Italy
* Correspondence: mariachiara.alterisio@unina.it; Tel.: +39-0812536001

**Table S2:** The table summarizes the clinical udder health status based on overall bacteriological culture results. It presents data from assessments conducted according to the mastitis management protocol used on the farm. The table differentiates cases with clinical signs (clinical mastitis, CM) and those without clinical signs (subclinical mastitis, SCM). The data are drawn from the comprehensive dataset of cows, including those receiving the complementary feed via an intraruminal slow-release bolus and those that did not.

| **Clinical assessment of udder health** | | |
|---|---|---|
| **Status** | **No** | **%** |
| **CM** | 27 | 12.1 |
| **SCM** | 197 | 87.9 |
| **Overall BMC performed** | | |
| **Status** | **No** | **%** |
| **CM** | 27 | 100.0 |
| **SCM** | 102 | 51.8 |
| **Overall BMC results** | | |
| **Status CM** | **No** | **%** |
| *Escherichia coli* | 5 | 18.5 |
| *Staphylococcus aureus* | 9 | 33.3 |
| *Staphylococcus chromogens* | 2 | 7.4 |
| *Streptococcus dysgalactie* | 5 | 18.5 |
| *Staphylococcus hemolyticus* | 5 | 18.5 |
| *Streptcoccus uberis* | 1 | 3.7 |
| **Status SCM** | **No** | **%** |
| *Corynebacterium bovis* | 9 | 8.8 |
| *Enterococcus faecalis* | 5 | 4.9 |
| *Escherichia coli* | 2 | 2.0 |
| *Staphylococcus aureus* | 42 | 41.2 |
| *Sthapylococcus chromogens* | 11 | 10.8 |
| *Streptococcus dysgalactie* | 4 | 3.9 |
| *Sthapylococcus epidermidis* | 4 | 3.9 |
| *Sthapylococcus hemolyticus* | 19 | 18.6 |
| *Staphylococcus simulans* | 6 | 5.9 |

BMC=bacteriological milk culture; No=number; %=percentage
